# Contactless doping characterization of ${\mathrm{Ga}_{2}\mathrm{O}_{3}}$ using acceptor Cd probes

**DOI:** 10.1038/s41598-022-18121-y

**Published:** 2022-08-26

**Authors:** Marcelo B. Barbosa, João Guilherme Correia, Katharina Lorenz, Armandina M. L. Lopes, Gonçalo N. P. Oliveira, Abel S. Fenta, Juliana Schell, Ricardo Teixeira, Emilio Nogales, Bianchi Méndez, Alessandro Stroppa, João Pedro Araújo

**Affiliations:** 1 IFIMUP, Institute of Physics for Advanced Materials, Nanotechnology and Photonics, Departamento de Física e Astronomia da Faculdade de Ciências da Universidade do Porto, Rua do Campo Alegre 687, 4169-007 Porto, Portugal; 2 Department of Physics, Faculty of Science, National University of Singapore, 2 Science Drive 3, Singapore, 117542 Singapore; 3 C2TN, DECN, Instituto Superior Técnico, Universidade de Lisboa, Bobadela, Portugal; 4 EP Department, European Organization for Nuclear Research (CERN), 1211 Geneva, Switzerland; 5 INESC-MN, IPFN, Instituto Superior Técnico, Universidade de Lisboa, Lisbon, Portugal; 6 Physics Department and CICECO, University of Aveiro, 3810-193 Aveiro, Portugal; 7 KU Leuven, Instituut voor Kern- en Stralingsfysica, Celestijnenlaan 200 D, 3001 Leuven, Belgium; 8 Institute for Materials Science and Center for Nanointegration Duisburg-Essen (CENIDE), University of Duisburg-Essen, 45141 Essen, Germany; 9 Departamento de Física de Materiales, Universidad Complutense de Madrid, 28040 Madrid, Spain; 10 CNR-SPIN c/o Università degli Studi dell’Aquila, Via Vetoio 10, 67010 Coppito, L’Aquila Italy

**Keywords:** Electronic properties and materials, Semiconductors, Characterization and analytical techniques

## Abstract

Finding suitable p-type dopants, as well as reliable doping and characterization methods for the emerging wide bandgap semiconductor $\beta$-${\mathrm{Ga}_{2}\mathrm{O}_{3}}$ could strongly influence and contribute to the development of the next generation of power electronics. In this work, we combine easily accessible ion implantation, diffusion and nuclear transmutation methods to properly incorporate the Cd dopant into the $\beta$-${\mathrm{Ga}_{2}\mathrm{O}_{3}}$ lattice, being subsequently characterized at the atomic scale with the Perturbed Angular Correlation (PAC) technique and Density Functional Theory (DFT) simulations. The acceptor character of Cd in $\beta$-${\mathrm{Ga}_{2}\mathrm{O}_{3}}$ is demonstrated, with Cd sitting in the octahedral Ga site having a negative charge state, showing no evidence of polaron deformations nor extra point defects nearby. The possibility to determine the charge state of Cd will allow assessing the doping type, in particular proving p-type character, without the need for ohmic contacts. Furthermore, a possible approach for contactless charge mobility studies is demonstrated, revealing thermally activated free electrons for temperatures above $\sim$ 648 K with an activation energy of 0.54(1) and local electron transport dominated by a tunneling process between defect levels and the Cd probes at lower temperatures.

## Introduction

${\mathrm{Ga}_{2}\mathrm{O}_{3}}$ is a wide band gap semiconductor of growing interest due to its potential application in power and high-voltage electronic devices^1–4^. It is transparent in the ultraviolet (UV) range, thus being also very promising for solar blind UV optoelectronic devices^3,5–7^. With a band gap of 4.8 eV^8^, $\beta$-${\mathrm{Ga}_{2}\mathrm{O}_{3}}$, the most stable of its polymorphic forms, is a large gap insulator, but its conductivity actually depends on doping and growth conditions^1,3^. N-type semiconductivity is often observed and commonly attributed to ionized oxygen vacancies which act as donors^1,9^, but after first-principles calculations have revealed that oxygen vacancies act as deep donors in ${\mathrm{Ga}_{2}\mathrm{O}_{3}}$ (thus cannot be responsible for electron conductivity), Varley et al.^5,7^ questioned this assumption attributing it to background impurities such as H, Si and Ge. Concerning p-type doping, several potential candidates have been proposed, such as Cd^10^, Fe^11,12^, Mg^12–14^ and N^14,15^, as well as Ga vacancies without oxygen vacancy compensation in undoped ${\mathrm{Ga}_{2}\mathrm{O}_{3}}$^16,17^. Good p-type carrier concentration has been observed for N-doped ${\mathrm{Ga}_{2}\mathrm{O}_{3}}$ thin films in the context of potential solar blind photo-responsive applications^18,19^ and, in other works, p-type conductivity has even been reported but carrier mobility and concentrations have not been analyzed or have been revealed too low for practical applications^4,16,17,20,21^. On the other hand, theorists pointed out fundamental issues for p-type doping due to potentially strong hole localization in ${\mathrm{Ga}_{2}\mathrm{O}_{3}}$ limiting hole mobility^22^. In fact, recent DFT studies indicate that such behavior is typical for cation-site impurities such as group 2 (e.g. Mg, Ca) and group 12 (e.g. Zn, Cd) acceptors which furthermore exhibit deep acceptor levels^23^. Moreover, the most studied acceptor, Mg, was shown to be incorporated not only in substitutional but also in interstitial Ga-sites as well as in the substitutional O-site where it acts as compensating donor^24^. Nevertheless, a cathodoluminescence study on Zn-doped ${\mathrm{Ga}_{2}\mathrm{O}_{3}}$ suggests a relatively shallow acceptor level 0.26 eV above the valence band maximum^25^, underlining the importance of further experimental studies concerning p-type doping. Furthermore, the charge mobility in doped samples is not always easily probed, specially for small-scale samples where depositing ohmic contacts is not always possible.

Considering the case of Cd-doped $\beta$-${\mathrm{Ga}_{2}\mathrm{O}_{3}}$, previous works report evidence of Cd located in substitutional Ga sites in $\beta$-${\mathrm{Ga}_{2}\mathrm{O}_{3}}$^10,26,27^, thus to better understand the concrete potentialities of p-type doping using Cd, in this work we identified the atomic scale location and charge state of implanted/diffused Cd probes in $\beta$-${\mathrm{Ga}_{2}\mathrm{O}_{3}}$. Then, we studied the charge mobility in this material using a contactless method that is here proposed as a possible solution to determine the n-type or p-type character of a wide variety of ${\mathrm{Ga}_{2}\mathrm{O}_{3}}$ samples containing different dopants. These studies were performed using the Perturbed Angular Correlation technique (PAC)^28–31^. It accurately measures the electric field gradient (EFG) due to the surrounding charge distribution of a probe nucleus, thus being a unique tool to study the location of impurities, as well as their charge states and interaction with defects. EFG is a traceless diagonal tensor fully characterized by its $V_{zz}$ component and the axial asymmetry parameter $\eta = \left( V_{xx}-V_{yy}\right) /V_{zz}$, where the observable frequency is $\omega _0 \propto eQV_{zz}$ (see Supplementary Information for more details), with *e* being the elementary charge and *Q* the probes’ quadrupole moment^28^.

## Results

### PAC experiments

Two different radioactive probe elements were used, ${}^{111m}$Cd and ${}^{111}$In, both decaying to stable ${}^{111}$Cd by gamma-gamma ($\gamma$-$\gamma$) emission through the same spin 5/2+, 245 keV probe level of ${}^{111}$Cd^32^.

**Figure 1:**
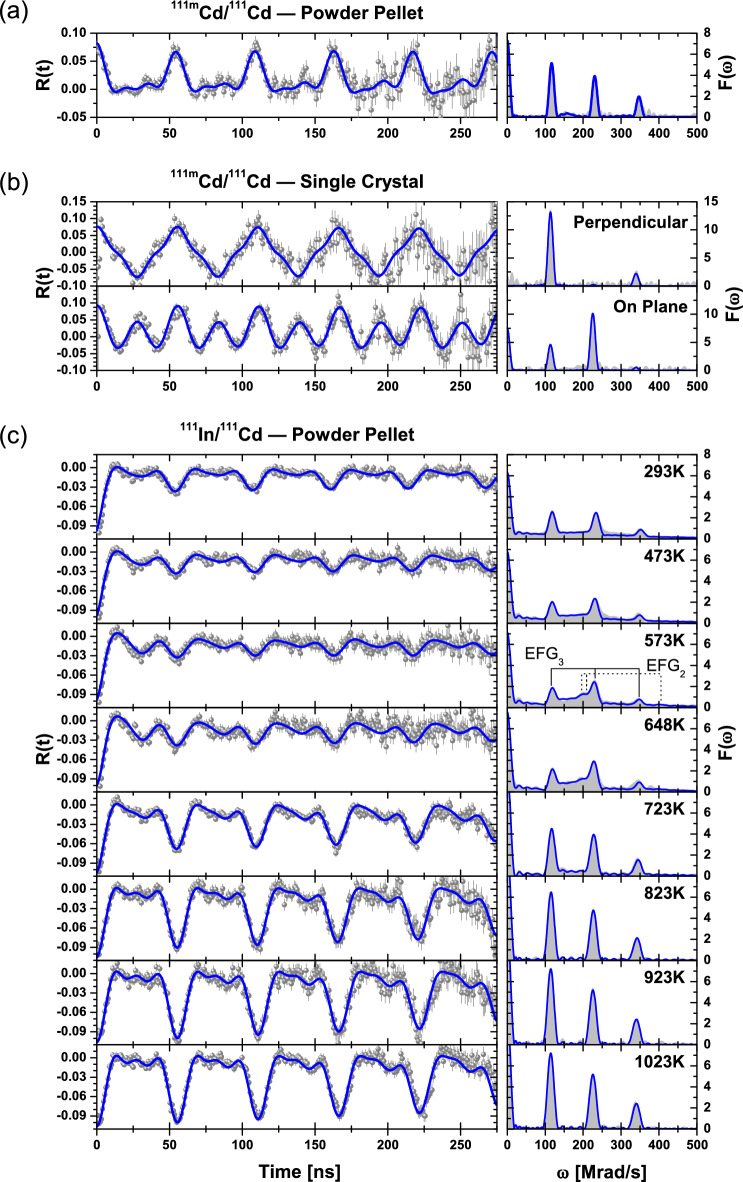
PAC spectra (symbols) and their fits (lines) (as well as their Fourier transforms) after implantation of ${}^{111m}$Cd (**a**) in the powder pellet and (**b**) in the single crystal, and (**c**) as a function of temperature after diffusion of $^{111}$In in the powder pellet. The triplet of frequencies associated with the EFGs of the intermediate (2) and final (3) states are shown in the Fourier transform at ${573}{ \,\mathrm{K}}$.

${}^{111m}$Cd, with no element transmutation, was used to directly study the location of the implanted dopants. Room temperature implantation into powder pellet and single crystal samples was performed (see Supplementary Information for more details) at ISOLDE-CERN^33^ to a low fluence of ${10^{11}}$atoms/cm$^2$. After subsequent 10 min air annealing at 1473  K and at 1273 K, respectively, the measurements were carried out at room temperature using a standard PAC analog spectrometer^34^ (for the single crystal a subset of 4 detectors was used, considering two different surface normal orientations with respect to the detectors plane). For nuclear level spin 5/2, a triplet of frequencies characterizes each EFG at the PAC observable R(t) function and respective Fourier transform. In the powder pellet, the experiments showed a dominant position for the Cd probes with an EFG characterized by $\omega _0 = {115.5}{\,\mathrm{Mrad}/\mathrm{s}}$, $\eta = {0.11}$ (Fig. 1a). However, $21\%$ of the probes were still in a defective environment (probably due to grain boundaries), while the equivalent experiments done in the single crystal showed a similar EFG ($\omega _0 = {112.9}\,{\mathrm{Mrad}/\mathrm{s}}$, $\eta = {0.09}$) but with 100% of the probes incorporated on undisturbed substitutional sites (Fig. 1b).

In the case of ${}^{111}$In, the probe decays by electron capture of an inner atomic K- or L-shell electron, leaving ${}^{111}$Cd in an ionized unstable state before the nuclear $\gamma$-$\gamma$ emission occurs. The first inner hole created at the restructuring Cd atomic shells is very rapidly recovered (within about $10^{-14}$ s) by electrons of higher orbits with a consequent emission of X-rays and/or Auger electrons, (leading to further ionization of the atom), which loses on average 3 to 8 electrons^35,36^. While reaching stability, the charge distribution around the probe (hence the EFG) is changing, thus monitoring this EFG by PAC as a function of temperature enables studying the formation (and annihilation) of ionized and excited electronic states of Cd. Here, for the analysis of fluctuating EFGs, the theory of stochastic processes applied to dynamic transitions in PAC^37^ is necessary. It assumes that the environment around a probe might change with time in such a way that allowed transitions between different states occur in a “Markov chain”-like fashion. Therefore, the system is described by the EFGs that characterize each possible state and by the transition rates to go from one state to another. In this context, the experimental data was analyzed using a first principles fitting program integrating multiple EFG states and their mutual transition rates^38^.

The powder pellet sample was wetted with a ${}^{111}$In (aq) solution, dried and annealed in air during 48 hours at 1373  K for promoting ${}^{111}$In diffusion. Afterwards, PAC measurements were performed between 293 K and 1023 K (Fig. 1c). Room temperature (293 K) control measurements were performed before and after the full series of experiments. No observable changes were present ensuring that no irreversible annealing effects occurred during the experiments.

The spectra at 923 K and at 1023 K are indistinguishable with EFG signature characterized by $\omega _0 = {113}\,{\mathrm{Mrad}/\mathrm{s}}$ and $\eta = {0.10}$. These results present a perfect match with the EFG obtained from the ${}^{111m}$Cd measurements. That same EFG is visible at all temperatures but the amplitude of the corresponding R(t) spectra clearly varies. Since the PAC measurements are reversible with temperature, and there are no structural $\beta$-${\mathrm{Ga}_{2}\mathrm{O}_{3}}$ phase transitions in this range of temperatures^1^, the observed effects strongly point to a temperature dependence of the Cd electronic recovery after electron capture. In particular, directly after the In decay, and before sufficient electronic recovery is achieved, the charge distribution around a Cd atom is variable and uncertain. This leads to an initial state described by a broad EFG distribution with central EFG ($\omega _0 = {111}\,{\mathrm{Mrad}/\mathrm{s}}$, $\eta = {1.0}$) and $\text {FWHM} > {30}\,{\mathrm{Mrad}/\mathrm{s}}$. Then, a unidirectional transition occurs to a final stable state characterized by the EFG mentioned earlier.

At high temperatures the electronic recovery is faster than the experimentally observable timescale, where only the final stable configuration is observed. Furthermore, all probes are found in a single location and the low damping $(\text {FWHM} = {1.4}\,{\mathrm{Mrad}/\mathrm{s}})$ suggests that the concentration of point defects in the next-nearest lattice neighborhood are much below the $\sim$1 ppm probe’s concentration.

At 573 K and 648 K a meta-stable Cd state was also observed (intermediate state), characterized by $\omega _0 = {117}{\mathrm{Mrad}/\mathrm{s}}$, $\eta = {0.92}$ and very low $\text {FWHM} < {2}{\mathrm{Mrad}/\mathrm{s}}$. At these temperatures, this meta-stable electronic state of Cd has ${\sim }7\%$ probability of being formed before achieving the final state. Due to the large $\eta$, the first two observable triplet frequencies are very close, merging into a single broader peak around ${200}\,{\mathrm{Mrad}/\mathrm{s}}$ in the Fourier spectra. Moreover, it was observed that the EFG of every state barely changes with temperature while the transition rates ($R_{i \rightarrow j}$) have strong variations, in particular for the transition $R_{1 \rightarrow 3}$ between the initial ($i=1$) and final ($j=3$) states above 648 K (see Fig. 3).

### DFT simulations

**Figure 2:**
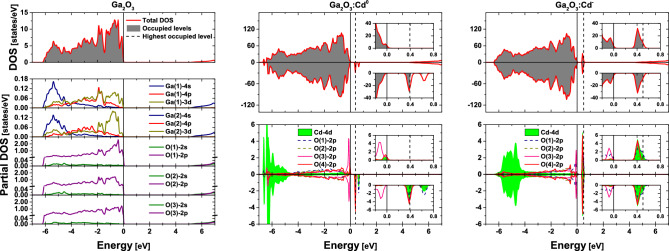
Total and atom-resolved partial density of states (DOS) of ${\mathrm{Ga}_{2}\mathrm{O}_{3}}$ and supercells of (${\mathrm{Ga}_{2}\mathrm{O}_{3}}$:Cd)^0^ and (${\mathrm{Ga}_{2}\mathrm{O}_{3}}$:Cd)^−^. The top valence band of ${\mathrm{Ga}_{2}\mathrm{O}_{3}}$ is set at 0 eV (full line) and the energy of the highest occupied level is represented by a dashed line. For pure ${\mathrm{Ga}_{2}\mathrm{O}_{3}}$, both lines are coincident. The insets show a zoomed energy range focused on the impurity band. Partial DOS includes orbital contribution from all non-equivalent atoms of ${\mathrm{Ga}_{2}\mathrm{O}_{3}}$, as well as the 4*d* contribution of Cd and the 2*p* contribution of its neighboring O atoms (due to symmetry break, Cd has 4 non-equivalent O neighbors instead of 3).

The PAC results were compared to DFT calculations as implemented in the WIEN2k package^39^ (see Supplementary Information for more details). A unit cell of $\beta$-${\mathrm{Ga}_{2}\mathrm{O}_{3}}$ was considered using the lattice parameters reported by Åhman et al.^40^. The unit cell has a sixfold coordinated Ga site (octahedral), a fourfold coordinated Ga site (tetrahedral) and three non-equivalent O sites. After structural relaxation, a band gap of 4.91 eV was obtained from the total density of states (DOS) (Fig. 2) in good agreement with the experimental value of 4.85 eV^1^. Moreover, the atom-resolved partial density of states (PDOS) shows a very small contribution from the Ga-3*d*, Ga-4*s* and Ga-4*p* orbitals to the upper valence band that is completely dominated by the O-2*p* orbitals, whereas the conduction band minimum is mainly described by the Ga-4*s* orbitals (Fig. 2).

From the occupancy of In atoms in similar ${\mathrm{InGaO}_3}$ and ${\mathrm{In}_2\mathrm{O}_3}$ structures^26^, it was expected that both In (diffused) and Cd (implanted)^10^ incorporate into the larger octahedral Ga site in ${\mathrm{Ga}_{2}\mathrm{O}_{3}}$. Therefore, a ${1\times 4\times 2}$ supercell of ${\mathrm{Ga}_{2}\mathrm{O}_{3}}$ was constructed having a Cd atom placed in an octahedral Ga site (1:64 dilution). Since Ga has 3 valence electrons whereas Cd has only 2, two different charge states were considered for the Cd probe: neutral (${\mathrm{Cd}^{0}}$) and charged (${\mathrm{Cd}^{-}}$), the latter by adding one extra electron to the lattice which is compensated by an additional homogeneous positive background to keep the entire cell in a neutral state^41,42^. The EFGs at the Cd probe were calculated for each configuration (Table 1) and a very good agreement was found between the EFGs for ${\mathrm{Cd}^{-}}$ and the final state in the PAC experiments, as well as between the EFGs for ${\mathrm{Cd}^{0}}$ and the intermediate state observed at 573  K (Table S2 in the Supplementary Information contains the calculated EFGs for other Cd sites and charge states).

**Table 1:** $V_{zz}$ and $\eta$ for each simulated Cd charge state and PAC experimental states.

	$V_{zz}$ (V/Å$^2$)	$\eta$
${\mathrm{Ga}_{2}\mathrm{O}_{3}}$:${\mathrm{Cd}^{0}}$	70.2	0.98
${\mathrm{Ga}_{2}\mathrm{O}_{3}}$:${\mathrm{Cd}^{-}}$	63.8	0.04
Exp. initial state	64(2)	1.0(1)
Exp. intermediate state	67(2)	0.92(3)
Exp. final state	65(1)	0.10(1)

Experimental values of $V_{zz}$ were estimated from the experimental frequencies using the electric quadrupole moment $Q = {0.765(15)}{\,\mathrm{b}}$^43^.

**Table 2:** *p*, *d* and *s*-*d* valence contributions to the EFG tensor in the principal components axes (in units of V/Å$^{2}$) for each Cd charge state (by definition, $\left| V_{zz} \right| \ge \left| V_{yy} \right| \ge \left| V_{xx} \right|).$

	${\mathrm{Ga}_{2}\mathrm{O}_{3}}$:${\mathrm{Cd}^0}$			${\mathrm{Ga}_{2}\mathrm{O}_{3}}$:${\mathrm{Cd}^{-}}$		
	$V_{xx}$	$V_{yy}$	$V_{zz}$	$V_{xx}$	$V_{yy}$	$V_{zz}$
p	− 19.6	− 24.4	43.9	− 23.1	− 28.6	51.7
d	18.3	− 46.0	27.7	− 7.9	− 5.0	12.9
s-d	0.5	0.8	− 1.3	0.6	0.7	− 1.4

The total DOS shows that replacing a Ga atom by a ${\mathrm{Cd}^0}$ probe induces a partially filled impurity band in the band gap near the top valence band while the basic electronic structure of ${\mathrm{Ga}_{2}\mathrm{O}_{3}}$ remains unaltered (Fig. 2). By integrating the unfilled region of the band, a value of 1.00 electron is obtained, in accordance with the expected behavior of a Cd dopant with single-acceptor character in this material. By looking at the partial DOS projected at the Cd and O atoms (Fig. 2), it is possible to see that the impurity band is composed of Cd-4*d* and O-2*p* orbitals. A similar behavior is observed for ${\mathrm{Cd}^{-}}$, but in this case the impurity band gets completely filled. The top of the impurity band is $\sim$ 4 eV from the conduction band, which is very close to the 4.3 eV reported for the band gap of amorphous Cd-Ga-O thin films^44^. The analysis of the *p*, *d* and *s*-*d* valence contributions to the EFG tensor principal components ($V_{xx}, V_{yy}, V_{zz}$) for each Cd state (Table 2) reveals that the biggest variation occurs in the *d* valence contribution, mainly in the $V_{xx}$ and $V_{yy}$ components. This is in good agreement with the DOS results, since the impurity band is composed of Cd-4*d* states that get filled when going from the ${\mathrm{Cd}^0}$ to the ${\mathrm{Cd}^{-}}$ charge state. Consequently, relevant changes in $\eta$, which depends on $V_{xx}$ and $V_{yy}$, but small changes in $V_{zz}$ are expected for different charge states, i.e., in perfect agreement with both theoretical and experimental EFG values found for ${\mathrm{Cd}^0}$ and ${\mathrm{Cd}^{-}}$ (Table 1).

## Discussion

**Figure 3:**
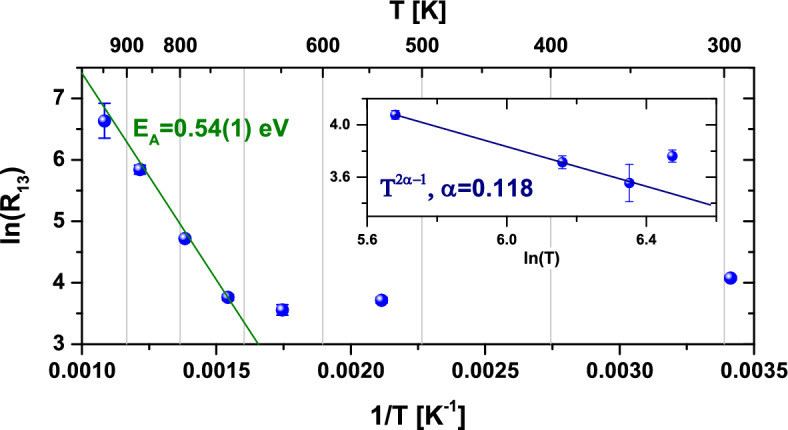
Arrhenius plot of the transition rate $R_{1 \rightarrow 3}$ (MHz) as a function of temperature in logarithmic scale and the corresponding fit between 648 K and 923 K. **(inset)** Logarithmic $R_{1 \rightarrow 3}$ plotted as a function of logarithmic temperatures between 293 K and 573 K. The linear curve corresponds to $T^{2\alpha -1}$ for $\alpha = 0.118$ (the slope of the curve is equal to $2\alpha -1$).

Having identified the position and charge states of Cd, the temperature dependence of the transition rates between the initial (electronically unstable) and the final (stable) states in the ${}^{111}$In PAC experiments provides insights about the electron mobility in ${\mathrm{Ga}_{2}\mathrm{O}_{3}}$. Our results revealed two different regimes (Fig. 3): (1) Above 648 K, the transition rates are described by an Arrhenius law with an activation energy of 0.54(1) eV. According to the present simulations, Cd induces an impurity band $\sim$ 0.4 eV above the top valence band of ${\mathrm{Ga}_{2}\mathrm{O}_{3}}$, which is tempting to attribute to this 0.54(1) eV activation energy. However, ${}^{111m}$Cd PAC measurements showed that, after annealing for removal of implantation defects, all Cd are found in the ${\mathrm{Cd}^{-}}$ charge state at room temperature, suggesting that the Fermi level lies above the acceptor level, as expected for n-type material. Fleischer and Meixner^45^ reported a thermal activation energy of 0.6(1) eV for carrier mobility in ${\mathrm{Ga}_{2}\mathrm{O}_{3}}$ and associated it with electron delocalization and increasing electron mobility with increasing temperature. Nonetheless, Ma et al.^46^ observed that charge carrier mobility and charge carrier density are interconnected, where ionized impurity scattering (which depends on donor concentration) decreases electron mobility. In addition, they identified polar optical phonon scattering as the dominant mechanism limiting electron mobility in $\beta$-${\mathrm{Ga}_{2}\mathrm{O}_{3}}$ by observing a decrease in electron mobility with increasing temperature (in contradiction to the temperature-dependence observed by Fleischer and Meixner^45^ but in accordance with the work of Oishi et al.^47^). Additionaly, deep level transient spectroscopy (DLTS) measurements performed by Irmscher et al.^48^ showed the presence of several deep defect levels inside the band gap, including one 0.55 eV and another 0.74 eV below the conduction band (the second being dominant). Similar results were found by Zhang et al.^49^, identifying defect levels 0.62 eV and 0.82 eV below the conduction band. Therefore, at the light of what is known, the activation energy of 0.54(1) eV obtained in this work is most likely associated with electrons going from defect levels (observed using DLTS^48,49^) to the conduction band, recalling the mentioned interconnection between electron mobility and donor concentration^46^. (2) Below 573  K, a slight decrease of $R_{1 \rightarrow 3}$ with rising temperature is, unexpectedly, observed. This counterintuitive phenomenon cannot be explained by low temperature conductive processes such as variable-range hopping. However, similar behavior is predicted for the quantum tunneling rate in certain regimes of a biased double-well potential coupled to a dissipative field with ohmic dissipation (the coupling field has ohmic dissipation if its spectral density is proportional to the frequency: $J(\omega ) \propto \omega$). For this reason, the hypothesis of direct electron tunneling from a defect to the Cd probe was explored assuming that the defect is in one of the potential wells and the Cd probe is in the other. The theory of such interaction has been developed in Refs.^50–52^ and applications can be found in the dynamics of single bistable defects coupled to an electron bath in disordered metals^53^ and in the tunneling of a Xe atom between the tip of a scanning-tunneling microscope (STM) or of an atomic-force microscope (AFM) and a Ni surface, coupled to phonons^54^. According to this theory, when $k_BT \gg \varepsilon$, where $\varepsilon$ is the bias energy between the two wells, the tunneling rate $\gamma$ between the wells is approximately:

1 $$\begin{aligned} \gamma\approx & {} \frac{\Delta _r}{2} \left( \frac{2\pi k_BT}{\hbar \Delta _r}\right) ^{2\alpha -1}\frac{\left| {\Gamma (\alpha )}\right| ^2}{\Gamma (2\alpha )} \end{aligned}$$

2 $$\begin{aligned} \ln (\gamma )\approx & {} \ln (\theta ) +\left( 2\alpha -1\right) \ln (T), \nonumber \\ \theta= & {} \frac{\Delta _r}{2} \left( \frac{2\pi k_B}{\hbar \Delta _r}\right) ^{2\alpha -1}\frac{\left| \Gamma (\alpha )\right| ^2}{\Gamma (2\alpha )} \end{aligned}$$

with $\Delta _r=\Delta _0 \left( \frac{\Delta _0}{\omega _p}\right) ^\frac{\alpha }{1-\alpha }$, where $\omega _p$ is the oscillation frequency in either well, $\Delta _0$ is the bare tunneling matrix element, $\Gamma (x)$ is the Gamma function and $\alpha$ is a dimensionless dissipation coefficient which is dependent on the material. In the regimes where $0< \alpha < 1/2$, the tunneling rate increases with decreasing temperature. Fitting the logarithmic equation to the transition rates obtained between 293 K and 573 K yields $\alpha = {0.118(4)}$ and $\Delta _r = {1.3(2)10^10} \mathrm{s}^{-1}$ (see inset of Fig. 3), hence we are in fact in the regime of $0< \alpha < 1/2$ and our transition rates behave as predicted by Eq. (1). The theory is only valid for a specific set of values for each variable, so we can test them and set boundaries in our case; firstly, $k_BT > \varepsilon$, thus $\varepsilon < {0.0252}\,\mathrm{eV}$. Then, the theory requires that $\hbar \Delta _r \ll \alpha k_BT$, which is true even at room temperature $\left( {8.53\times 10^{-6}} \,\mathrm{eV} \ll {2.98\times 10^{-3}}{ \mathrm{eV}}\right)$. The last requirement is that $\Delta _0 \ll \omega _p$. Considering as reference the frequency associated with the polar optical phonon energy reported in Ref.^46^ $(\omega _p={6.7(6)\times 10^{13}} s^{-1})$ and using the definition of $\Delta _r$, we have $\Delta _0 = {3.6(6)\times 10^{10}}\,s^{-1}$, therefore $\Delta _0 \ll \omega _p$ is verified. A hypothesis for the observations at lower temperatures can then be formulated, stating that electrons are tunneling from some defects directly to the Cd probes and that such a system can be described by a double-well potential coupled to a dissipative field with ohmic dissipation. In this case, we believe that phonons are the dissipative field, as observed in the example above from Louis et al.^54^. Although Leggett et al.^55,56^ suggest a super-ohmic dissipation ($J(\omega ) \propto \omega ^s$, with $s > 1$) in the case of defect (or electron) tunneling in a solid with coupling to a (three-dimensional) acoustic phonon bath, in our understanding the authors refer to random diffusion by quantum tunneling (implying several tunneling “jumps”), whereas we observe solely (single) direct tunneling of an electron from a specific defect to the Cd probe, thus the possibility of ohmic dissipation remains acceptable. Therefore, we propose that the lower temperature regime of the observed electronic recovery is explained by direct electron tunneling from defects to the Cd probes.

Concerning the issue of strong hole localization in ${\mathrm{Ga}_{2}\mathrm{O}_{3}}$ referred previously^22^, since no holes are present around the Cd probes, no polarons are formed in their vicinity (they would be readily observable in the PAC measurements by leading to different signature EFG values). Besides, the Cd-O bonds showed a more covalent character, in contrast to the ionic character observed for the Ga-O bonds (Fig. S3 in Supplementary Information), so the probability of polaron formation in an adjacent O atom is reduced. Nonetheless, it must be stressed that conclusions can only be made for the direct vicinity of the active probe atoms since the EFG decays with $1/r^3$ (with *r* being the distance from the defect causing the EFG).

Lastly, in the absence of experimental values, the ionization energy of the Cd acceptor has been estimated in previous DFT studies^23,24^ suggesting a thermodynamic transition level for ${\mathrm{Cd}^{0}}$/${\mathrm{Cd}^{-}}$ in ${\mathrm{Ga}_{2}\mathrm{O}_{3}}$ with a deep level character ($\sim$ 1 eV). If such a high level is confirmed experimentally, Cd would be unsuitable as acceptor for efficient p-type doping. Still, looking forward for extended studies of differently pre-doped, electrically characterized, ${\mathrm{Ga}_{2}\mathrm{O}_{3}}$ samples, the present research method could provide detailed insight of the role of co-dopant Cd atoms in a more complex and tunable doping scheme. Also, it must be specially stressed that the used method enabled the study of charge mobility in ${\mathrm{Ga}_{2}\mathrm{O}_{3}}$ samples without the use of ohmic contacts (which are not always easily produced, in particular for small samples). In fact, since the acceptor character of Cd is observed in the transient measurements but the relaxed sample has no free holes available due to its concluded intrinsic n-type character and extremely low concentration of Cd ions (order of $10^{10} \,{\mathrm{ions}/\mathrm{cm}^{2}}$), alternative methods such as Hall measurements would show no clues of the Cd acceptor character in the samples here reported, thus showcasing the potential use of the method here presented on samples whose doping concentration is too low to change the macroscopic doping character of the semiconductor but the impurity character in the material is wished to be probed.

## Conclusion

In summary, by comparing the EFG at Cd impurity sites obtained in PAC experiments with DFT simulations we have confirmed that Cd occupies a single position in $\beta$-${\mathrm{Ga}_{2}\mathrm{O}_{3}}$, the substitutional octahedral Ga site, and it shows a clear acceptor character. Two Cd charge states were observed, the neutral unstable ${\mathrm{Cd}^0}$ with a short mean life time ($\frac{1}{R_{23}} \approx {2} \;\upmu \mathrm{s}$ at 573 K, see the Supplementary Information) and the stable ionized acceptor ${\mathrm{Cd}^{-}}$. The observation of the electronic recovery after electron capture decay of ${}^{111}$In onto ${}^{111}$Cd (which results in a evolution of the EFG at the Cd site during the PAC experiments) enabled the identification of two regimes with distinct transport properties: above 648 K the electron mobility and/or density increases with increasing temperature with an activation energy of 0.54(1) eV. For lower temperatures, surprisingly the local conductivity seems to decrease with increasing temperature which is attributed to an electron tunnelling mechanism. It was shown that the use of very accessible ${}^{111}$In activated solutions (currently used in hospitals as tracers) might be a feasible option to properly dope a $\beta$-${\mathrm{Ga}_{2}\mathrm{O}_{3}}$ sample with Cd by transmutation. Finally, the experimental signature of the different charge states of Cd reported here can provide an easy and contactless method to determine the n-type or p-type character of a wide variety of ${\mathrm{Ga}_{2}\mathrm{O}_{3}}$ samples containing different dopants.

### Supplementary Information

Supplementary Information.

## Data Availability

All data generated or analysed during this study are included in this published article (and its Supplementary Information files).
