# Author Correction: Contactless doping characterization of Ga_2_O_3_ using acceptor Cd probes

**DOI:** 10.1038/s41598-024-56764-1

**Published:** 2024-03-20

**Authors:** Marcelo B. Barbosa, João Guilherme Correia, Katharina Lorenz, Armandina M. L. Lopes, Gonçalo N. P. Oliveira, Abel S. Fenta, Juliana Schell, Ricardo Teixeira, Emilio Nogales, Bianchi Méndez, Alessandro Stroppa, João Pedro Araújo

**Affiliations:** 1 IFIMUP, Institute of Physics for Advanced Materials, Nanotechnology and Photonics, Departamento de Física e Astronomia da Faculdade de Ciências da Universidade do Porto, Rua do Campo Alegre 687, 4169-007 Porto, Portugal; 2 Department of Physics, Faculty of Science, National University of Singapore, 2 Science Drive 3, Singapore, 117542 Singapore; 3 C2TN, DECN, Instituto Superior Técnico, Universidade de Lisboa, Bobadela, Portugal; 4 EP Department, European Organization for Nuclear Research (CERN), 1211 Geneva, Switzerland; 5 INESC-MN, IPFN, Instituto Superior Técnico, Universidade de Lisboa, Lisbon, Portugal; 6 Physics Department and CICECO, University of Aveiro, 3810-193 Aveiro, Portugal; 7 KU Leuven, Instituut voor Kern- en Stralingsfysica, Celestijnenlaan 200 D, 3001 Leuven, Belgium; 8 Institute for Materials Science and Center for Nanointegration Duisburg-Essen (CENIDE), University of Duisburg-Essen, 45141 Essen, Germany; 9 Departamento de Física de Materiales, Universidad Complutense de Madrid, 28040 Madrid, Spain; 10 CNR-SPIN c/o Università degli Studi dell’Aquila, Via Vetoio 10, 67010 Coppito, L’Aquila Italy

Correction to: *Scientific Reports* 10.1038/s41598-022-18121-y, Published online 26 August 2022

The acknowledgements section in the original version of this article was incomplete, where a project funding number was omitted.

“This work was performed within the ISOLDE proposal IS481 and supported by FCT-Portugal, projects CERN-FP-123585-2011, CERN-FIS-PAR-0005-2017, CERN/FIS-TEC/0003/2019, POCI-01-0145-FEDER-032527 and PTDC/CTM-CTM/3553/2020, and by the European Commission through FP7- ENSAR (Contract 262010) and Horizon 2020 program ENSAR2 (Contract 654002). M. B. B. acknowledges a scholarship from FCT, SFRH/ BD/97591/2013, J. S. a grant from the Federal Ministry of Education and Research (BMBF), 05K16PGA. The authors further acknowledge E. G. Víllora and K. Shimamura (NIMS, Japan) for supplying the single crystal samples, the ISOLDE-CERN collaboration for supportive access to beam time and PD Reiner Vianden and the BONIS team at HISKP (Bonn, Germany) for the Cd implantations used for preliminary tests.”

Now reads:

"This work was performed within the ISOLDE proposal IS481 and supported by FCT-Portugal, projects CERN-FP-123585-2011, CERN-FIS-PAR-0005-2017, CERN/FIS-TEC/0003/2019, POCI-01-0145-FEDER-032527, PTDC/CTM-CTM/3553/2020 and UIDB/04349/2020, and by the European Commission through FP7- ENSAR (Contract 262010) and Horizon 2020 program ENSAR2 (Contract 654002). M. B. B. acknowledges a scholarship from FCT, SFRH/ BD/97591/2013, J. S. a grant from the Federal Ministry of Education and Research (BMBF), 05K16PGA. The authors further acknowledge E. G. Víllora and K. Shimamura (NIMS, Japan) for supplying the single crystal samples, the ISOLDE-CERN collaboration for supportive access to beam time and PD Reiner Vianden and the BONIS team at HISKP (Bonn, Germany) for the Cd implantations used for preliminary tests.”

The original article has been corrected.

