# Supplementary material for: Contactless doping characterization of Ga2O3 using acceptor Cd probes
Source: Sci Rep. 2022 Aug 26;12:14584. doi: 10.1038/s41598-022-18121-y (PMC9418202; doi:10.1038/s41598-022-18121-y)
Supplement: Supplementary file 1 — Supplementary Information. [file 41598_2022_18121_MOESM1_ESM.pdf]

Supplementary Information for
"Contactless doping characterization of Ga_2O_3 using acceptor Cd probes"

Marcelo B. Barbosa, João Guilherme Correia, Katharina Lorenz, Armandina M. L. Lopes, Gonçalo N. P. Oliveira, Abel S. Fenta, Juliana Schell, Ricardo Teixeira, Emilio Nogales, Bianchi Méndez, Alessandro Stroppa, João Pedro Araújo

PERTURBED ANGULAR CORRELATIONS

Theoretical Background

In a Perturbed Angular Correlation experiment, a radioactive probe which decays in a double cascade (emitting two photons, γ_1 and γ_2) is introduced in a sample by implantation, diffusion or neutron activation. The hyperfine interaction of the electric field gradient (EFG) at the probe's site with the electric quadrupole moment of the intermediate level of the cascade causes a time-dependent perturbation in the angular dependence of the emission probability of γ_2 with respect to γ_1. Since the EFG is a traceless matrix and diagonal in its principal axis, it can be completely described by only two parameters: the V_{zz} component and the axial asymmetry parameter $\eta = (V_{xx} - V_{yy})/V_{zz}$, considering that $|V_{xx}| \leq |V_{yy}| \leq |V_{zz}|$ [1, 2].

The time-dependent oscillations in the anisotropic emission of γ_2 then define the observable frequency ω_0 which is proportional to the quadrupole interaction frequency ω_Q

$$\omega_0 = k\omega_Q, \quad \omega_Q = \frac{eQV_{zz}}{4I(2I-1)\hbar} \tag{1}$$

where I and Q are the spin and the electric quadrupole moment of the intermediate level of the cascade, respectively, and $k = 3$ (or 6) for integer (or half-integer) spin [1, 2].

The coincidence spectra $N(\theta, t)$ can be recorded, where θ is the angle between detectors and t is the time delay between the detection of γ_1 and γ_2, allowing for the experimental perturbation function

$$R(t) = 2\frac{N(180°, t) - N(90°, t)}{N(180°, t) + 2N(90°, t)} \approx \sum A_{kk}G_{kk} \tag{2}$$

to be calculated, where A_{kk} are the angular correlation coefficients of the nuclear decay cascade and G_{kk} is the perturbation factor. For a polycrystalline sample, $G_{kk} = S_{k0} + \sum_n S_{kn} \cos\omega_n t$, so it is described by a sum of oscillatory terms with frequencies ω_n which correspond to transitions between the hyperfine levels created due to the splitting of the nuclear energy levels by the hyperfine interaction. The splitting of an intermediate level with spin $I = 5/2$ results in three sub-levels, so transitions between them will yield a triplet of frequencies ω_1, ω_2 and $\omega_3 = \omega_1 + \omega_2$, with $\omega_n = C_n(\eta)\omega_0$. Therefore, for each EFG present in the system, three peaks will be observed in the Fourier transform of the perturbation function.

In the case of fluctuating EFGs, such as during the recovery processes of the electronic environment after the loss of electrons from the lower atomic orbits due to electron capture decay, a dynamic description of PAC spectra is needed. Here, the theory based on stochastic processes applied to PAC developed by Winkler and Gerdau [3] was considered. In their formalism, the time-evolution operator changes from the usual $\hat{\Omega}(t) = \exp\left(-\frac{i}{\hbar}\hat{H}t\right)$ to $\hat{\Omega}(t) = \exp\left[\left(-\frac{i}{\hbar}H_{st}^{\times} + \hat{R}\right)t\right]$, where $\left(-\frac{i}{\hbar}H_{st}^{\times} + \hat{R}\right)$ is called the Blume matrix and $H_{st}^{\times}$ and $\hat{R}$ are Liouville operators, the former being constructed from the Hamiltonians that describe the different possible states (each one described by a static EFG) and the latter containing the transition rates between the different possible states (where the inverse of the sum of all transition rates from one state to the others corresponds to the mean life of that state). In this case, the observable perturbation factor is given by

$$G_{kk}(t) = \sum_{q=1}^{(2I+1)^2 N} a_{kq} \cos(\omega_q t) \exp(-\lambda_q t) \tag{3}$$

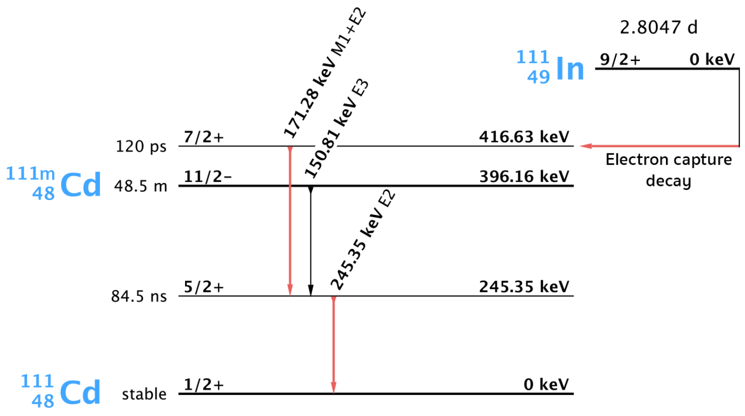

FIG. S1. Decay scheme for ^{111}In and ^{111m}Cd

where N is the number of possible states, the amplitudes a_{kq} depend on the eigenvectors of the Blume matrix and $-\lambda_q + i\omega_q$ are the eigenvalues. The real components of the eigenvalues are always negative and are only non-zero when the transition rates are also non-zero, thus the damping that they induce in the oscillations of the perturbation function are a signature of the presence of dynamic processes.

In this work, $N = 3$ different states were needed to describe the ^{111}In PAC experimental data, so the system is described by the ω_0, η and initial percentage at $t = 0$ of each individual state, plus the 6 transition rates between all pairs of states ($1 \to 2$, $1 \to 3$, $2 \to 1$, etc.), all of them acting as fitting parameters. Moreover, to account for the fact that probes in equivalent sites might have slight deviations in their EFGs (e.g., due to possible remaining diluted defects not preferentially attached to them), a Lorentzian distribution characterized by their central frequency ω_0 and full width at half maximum (FWHM) is integrated for each EFG individually in the fitting function.

The fitting is done by minimizing a chi-square function and the error of each fitting parameter is assumed to be the amount that they have to change in order for the chi-square function to vary one standard deviation.

Experimental Details

99.999% purity Ga_2O_3 powder was pressed into pellets of about 7 mm diameter and 2 mm thickness and subsequently annealed at 1773 K for 8 h. Single crystals were grown by the floating zone technique using 4N purity powder, then cut and polished in the (1 0 0) plane, as described elsewhere [4]. ^{111}In probes were introduced by wetting the powder pellet in a ^{111}In activated solution and annealed for 48 hours at 1373 K in air. The PAC measurements were performed as a function of the measurement temperature (between 293 K and 1023 K) in a 4-BaF_2 detector spectrometer [5] at CFNUL, in Lisbon. ^{111m}Cd probes were implanted with an energy of 30 keV in a powder pellet sample and in a single crystalline sample at ISOLDE/CERN to low fluences of 10^{11} atoms/cm^2 at room temperature. Then, the samples were annealed for 10 minutes at 1473 K and at 1273 K in air, respectively, in order to remove implantation defects. The PAC measurements were carried out at room temperature on a 6-BaF_2 detector spectrometer [5]. For the single crystal, two orientations were considered in a 4-detector's plane: one where the surface normal was perpendicular to the detector's plane and one in-plane at 45° from the detectors.

The decay scheme of both ^{111}In and ^{111m}Cd can be seen in Figure S1 and the fitting parameters from ^{111}In PAC as a function of temperature are summarized in Table S1.

DENSITY FUNCTIONAL THEORY SIMULATIONS

Simulation Details

The simulations were performed via the full-potential (linearized) augmented plane wave plus local orbitals [FP-(L)APW+lo] method as implemented in the WIEN2k code [6]. For the structural optimization (detailed below) and calculation of the electric field gradients, the generalized gradient approximation in the Perdew, Burke and Ernzerhof parameterization (GGA-PBE) [6] was considered as exchange-correlation functional. For the calculation of the density

TABLE S1. ^{111}In:Ga_2O_3 PAC fitting parameters. Three different states described by a single EFG each were considered. The transition rates between them are expressed in MHz, the quadrupole frequency ω_0 and the full width at half maximum for the Lorentzian, static-like, distribution (FWHM) are expressed in Mrad/s and the asymmetry parameter η is dimensionless.

T (K)	State 1 (initial)			State 2 (intermediate)			State 3 (final)			Transition Rates		
	ω_0	η	FWHM	ω_0	η	FWHM	ω_0	η	FWHM	$1 \rightarrow 2$	$1 \rightarrow 3$	$2 \rightarrow 3$
293	111(1)	1.0(1)	79(2)	-	-	-	117.8(1)	0.000(1)	1.4(3)	-	59(2)	-
473			66(2)	-	-	-	116.4(2)	0.000(2)		-	41(2)	-
573			47(8)	117(2)	0.92(3)	0(2)	116.1(1)	0.000(1)		3(1)	35(5)	0.5(1)
648			34(2)				115.7(2)	0.000(5)		3(2)	43(2)	8(4)
723			20(2)	-	-	-	114.6(1)	0.000(3)		-	112(3)	-
823			25(6)	-	-	-	113.7(1)	0.09(1)		-	344(26)	-
923			0(2)	-	-	-	113.1(1)	0.10(1)		-	761(216)	-

of states, band structure and band gap, the optimized structures were used and the modified Becke-Johnson exchange potential (mBJ) was applied, since it has been proven to better estimate band gaps in semiconducting materials than simply using GGA [7, 8]. It also has levels of agreement with experimental results comparable to hybrid functionals or Green function (GW) methods (which are computationally heavier and more time consuming) whilst being barely more expensive than GGA calculations [7, 8]. In fact, the current trend in the literature is that the mBJ is the best semilocal approximation to determine band gaps by achieving (on average) a better accuracy than hybrid functionals at a fraction of the computational cost [9].

The structural parameters of Ga_2O_3 in the β-phase (β-Ga_2O_3), as found in the work of Åhman *et al.* [10], were considered, i.e. $a = 12.214(3)$ Å, $b = 3.0371(9)$ Å, $c = 5.7981(9)$ Å, $\alpha = \gamma = 90°$ and $\beta = 103.83(2)°$, with the internal atomic positions being optimized by minimizing the atomic forces to a maximum limit of 2 mRy/bohr in a self-consistent way. Optimization of the lattice parameters using the very precise HSE06 hybrid functional was previously reported elsewhere [11] and the calculated lattice parameters are very close to the experimental ones (less than 0.5% variation), therefore, no lattice optimization was performed in this work and the experimental values were used for the simulations.

To simulate an isolated Cd impurity, a $1 \times 4 \times 2$ supercell of Ga_2O_3 with dimensions $a' = a = 12.214$ Å, $b' = 4b = 12.1484$ Å, $c' = 2c = 11.5962$ Å and $\beta = 103.83°$ was constructed. Its size was determined by increasing it until the variation of the EFG at the Cd site was in the same order of magnitude of the PAC experimental error.

A cut-off value for the plane wave expansion of Rmt*Kmax = 6.0 was considered, where Rmt is the muffin-tin sphere radius and Kmax is the largest K-vector of the plane wave expansion of the wave function. 90 and 20 k-points in the irreducible Brillouin zone were used for the Ga_2O_3 simple cell and for the $1 \times 4 \times 2$ supercell with the Cd impurity, respectively.

Different charge states for the Cd probes were considered, where additional charges were compensated by adding a homogeneous background of opposite charge to keep the entire cell in a neutral state [6, 12, 13]. For example, if an electron is added to the cell, the extra negative charge can be localized but a uniform positive charge will maintain the neutrality of the cell whilst not resulting in any extra interactions.

For the estimation of the thermodynamic transition level for Cd^0/Cd^-, the procedure employed in Refs. 14 and 15 was used, but the energy alignment in relation to bulk Ga_2O_3 was performed using the core energy levels from atoms far from the Cd probes instead of using the electrostatic potential.

Band Structure

The band structure of Ga_2O_3 (Fig. S2) shows an indirect band gap between the valence band maximum (VBM) located on the I-L line and the free-electron-like conduction band minimum (CBM) at the Gamma point. However, the valence band at the Gamma point is only 0.03 eV below that of the VBM, so there is an indirect band gap of 4.91 eV and a direct band gap of 4.94 eV, in good agreement with optical absorption measurements [16] and with previous calculations [11, 17]. By fitting the energy dispersion of the CBM at the Γ point to a parabolic function, an electron effective mass (m_e^*) of 0.35 m_e was obtained. This is close to the experimental value of 0.28 m_e [18] but slightly higher, which is expected since the used mBJ exchange potential generally overestimates the effective masses [19]. On the other hand, the top valence band is almost flat, indicating a rather large effective mass (m_h^*) for holes. This suggests that the electronic conductivity in Ga_2O_3 strongly depends on the mobility of the electrons that are thermally excited to the conduction band and less on the movement of holes created at the same time. These results

are consistent with previous reports [11, 17, 20–22].

In the band structure of the $1 \times 4 \times 2$ supercell with Cd^{-} in an octahedral Ga site (Fig. S2(b)), it is possible to see the induced impurity band (which is ∼0.4 eV above the top of the valence band) and that the top of the valence band remains very flat as in pure Ga_2O_3, thus the effective mass for holes remains very large.

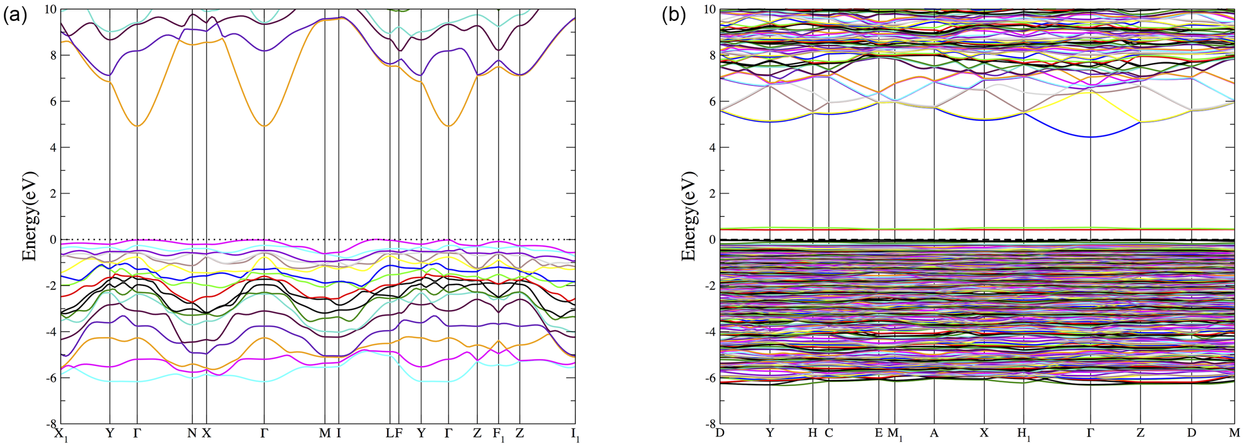

FIG. S2. Band structure of **(a)** pure Ga_2O_3 and **(b)** Cd^{-} in an octahedral Ga site of a $1 \times 4 \times 2$ supercell. The top valence band of pure Ga_2O_3 is set at 0 eV. The k-point labels are named as in Ref.[11] for pure Ga_2O_3 and as in Ref.[23] for the supercell containing Cd.

Electron Density

Fig. S3(a) shows that the electron density in pure Ga_2O_3 is highest around the O atoms (similar picture is observed in any cut-plane direction) thus hinting to an ionic-like character for the Ga-O chemical bonds.

In the supercell case containing Cd (Fig. S3(b)), it is possible to see that the charges are distributed between the Cd atom and its O atomic neighbors, indicating that the Cd-O bonds have a more covalent character in contrast to the ionic character exhibited by the Ga-O bonds for the Ga atoms in the same position.

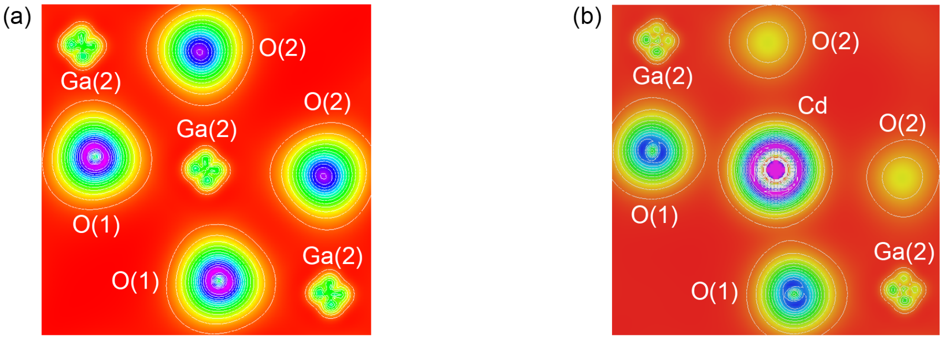

FIG. S3. Electron density considering the top of the valence band in a plane going through the centre of **(a)** Ga(2) and O(1) in pure Ga_2O_3 simple cell and of **(b)** O(1) and Cd^{-} in an octahedral Ga site within a $1 \times 4 \times 2$ supercell.

Electric Field Gradient

Besides the EFGs calculated for each Cd probe's charge state at the octahedral Ga site reported in the letter as matching the PAC experimental results, the EFGs for Cd probes at other sites and charge states were calculated as well. Each considered site is represented in Figure S4 and the resulting EFGs are gathered in Table S2.

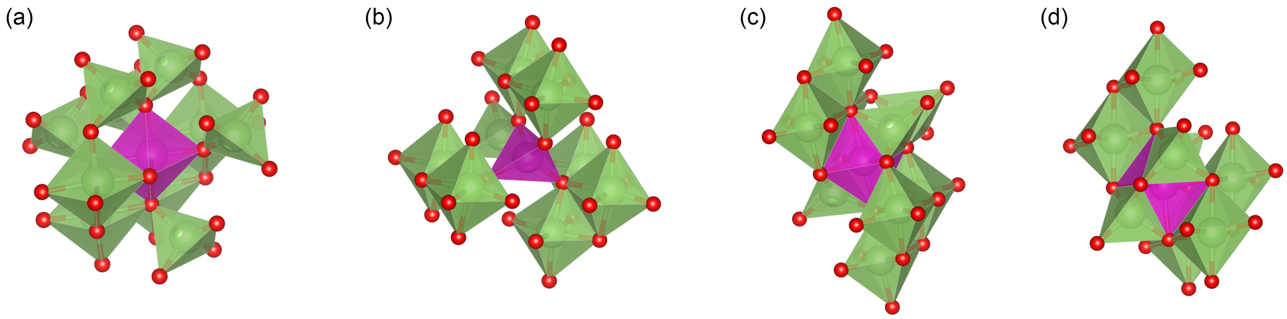

FIG. S4. Simulated Cd sites (in magenta) in supercells of β-Ga_2O_3: **(a)** octahedral Ga site, **(b)** tetrahedral Ga site, **(c)** interstitial (I) and **(d)** interstitial (II). The figures do not show the entire calculated supercells.

TABLE S2. Calculated V_{zz} and η for the Ga atoms in a simple cell of β-Ga_2O_3 and for each site (see Fig. S4) and charge state of the Cd probe in a $1 \times 4 \times 2$ supercell. The calculated distance to the oxygen nearest neighbors (ONN) is also shown.

	V_{zz} (V/Å^2)	η	ONN (Å)
Ga – octahedral	23.8	0.34	1.92 – 2.04
Ga – tetrahedral	-38.4	0.72	1.83 – 1.85
**Cd^0 – octahedral**	**70.2**	**0.98**	**2.11 – 2.25**
**Cd^- – octahedral**	**63.8**	**0.04**	**2.13 – 2.32**
Cd^0 – tetrahedral	-44.0	0.75	2.04 – 2.08
Cd^- – tetrahedral	-52.6	0.59	2.06 – 2.10
Cd^0 – interstitial (I)	38.7	0.45	2.17 – 2.20
Cd^{2+} – interstitial (I)	45.2	0.49	2.16 – 2.18
Cd^0 – interstitial (II)	-171.4	0.40	2.08 – 2.27
Cd^{2+} – interstitial (II)	-195.6	0.95	2.00 – 2.59

[1] G. Schatz and A. Weidinger, *Nuclear Condensed Matter Physics* (John Wiley and Sons, New York, 1996).
[2] T. Wichert, in *Identification of Defects in Semiconductors*, Semiconductors and Semimetals, Vol. 51, edited by M. Stavola (Elsevier, 1999) pp. 297 – 405.
[3] H. Winkler and E. Gerdau, Zeitschrift Fur Physik **262**, 363 (1973).
[4] E. G. Villora, K. Shimamura, Y. Yoshikawa, K. Aoki, and N. Ichinose, Journal of Crystal Growth **270**, 420 (2004).
[5] T. Butz, S. Saibene, T. Fraenzke, and M. Weber, Nuclear Instruments and Methods in Physics Research Section A: Accelerators, Spectrometers, Detectors and Associated Equipment **284**, 417 (1989).
[6] P. Blaha, K. Schwarz, G. K. H. Madsen, D. Kvasnicka, J. Luitz, R. Laskowski, F. Tran, and L. D. Marks, *WIEN2k: An Augmented Plane Wave plus Local Orbitals Program for Calculating Crystal Properties* (Vienna University of Technology, Austria, 2001).
[7] F. Tran and P. Blaha, Phys Rev Lett **102**, 226401 (2009).
[8] D. Koller, F. Tran, and P. Blaha, Physical Review B **83**, 195134 (2011).
[9] T. Rauch, M. A. L. Marques, and S. Botti, Journal of Chemical Theory and Computation **16**, 2654 (2020).
[10] J. Åhman, G. Svensson, and J. Albertsson, Acta Crystallographica Section C **52**, 1336 (1996).
[11] H. Peelaers and C. G. Van de Walle, Physica Status Solidi B-Basic Solid State Physics **252**, 828 (2015).
[12] P. E. Blochl, The Journal of Chemical Physics **103**, 7422 (1995).
[13] G. N. Darriba, M. Rentería, H. M. Petrilli, and L. V. C. Assali, Phys. Rev. B **86**, 075203 (2012).
[14] J. L. Lyons, Semiconductor Science and Technology **33**, 05LT02 (2018).
[15] H. Peelaers, J. L. Lyons, J. B. Varley, and C. G. Van de Walle, APL Materials **7**, 022519 (2019).
[16] H. H. Tippins, Phys. Rev. **140**, A316 (1965).
[17] A. Ratnaparkhe and W. R. L. Lambrecht, Applied Physics Letters **110**, 132103 (2017).
[18] M. Mohamed, C. Janowitz, I. Unger, R. Manzke, Z. Galazka, R. Uecker, R. Fornari, J. R. Weber, J. B. Varley, and C. G. Van de Walle, Applied Physics Letters **97** (2010), Artn 211903 10.1063/1.3521255.
[19] Y.-S. Kim, M. Marsman, G. Kresse, F. Tran, and P. Blaha, Phys. Rev. B **82**, 205212 (2010).
[20] J. B. Varley, J. R. Weber, A. Janotti, and C. G. V. d. Walle, Applied Physics Letters **97**, 142106 (2010).
[21] J. B. Varley, A. Janotti, C. Franchini, and C. G. Van de Walle, Physical Review B **85**, 081109(R) (2012).
[22] F. Litimein, D. Rached, R. Khenata, and H. Baltache, Journal of Alloys and Compounds **488**, 148 (2009).

[23] W. Setyawan and S. Curtarolo, Computational Materials Science **49**, 299 (2010).
